# Early MEK1/2 Inhibition after Global Cerebral Ischemia in Rats Reduces Brain Damage and Improves Outcome by Preventing Delayed Vasoconstrictor Receptor Upregulation

**DOI:** 10.1371/journal.pone.0092417

**Published:** 2014-03-18

**Authors:** Sara Ellinor Johansson, Stine Schmidt Larsen, Gro Klitgaard Povlsen, Lars Edvinsson

**Affiliations:** 1 Department of Clinical Experimental Research, Glostrup Research Institute, Glostrup Hospital, Glostrup, Denmark; 2 Division of Experimental Vascular Research, Department of Clinical Sciences, Lund University, Lund, Sweden; School of Pharmacy, Texas Tech University HSC, United States of America

## Abstract

**Background:**

Global cerebral ischemia following cardiac arrest is associated with increased cerebral vasoconstriction and decreased cerebral blood flow, contributing to delayed neuronal cell death and neurological detriments in affected patients. We hypothesize that upregulation of contractile ET_B_ and 5-HT_1B_ receptors, previously demonstrated in cerebral arteries after experimental global ischemia, are a key mechanism behind insufficient perfusion of the post-ischemic brain, proposing blockade of this receptor upregulation as a novel target for prevention of cerebral hypoperfusion and delayed neuronal cell death after global cerebral ischemia. The aim was to characterize the time-course of receptor upregulation and associated neuronal damage after global ischemia and investigate whether treatment with the MEK1/2 inhibitor U0126 can prevent cerebrovascular receptor upregulation and thereby improve functional outcome after global cerebral ischemia. Incomplete global cerebral ischemia was induced in Wistar rats and the time-course of enhanced contractile responses and the effect of U0126 in cerebral arteries were studied by wire myography and the neuronal cell death by TUNEL. The expression of ET_B_ and 5-HT_1B_ receptors was determined by immunofluorescence.

**Results:**

Enhanced vasoconstriction peaked in fore- and midbrain arteries 3 days after ischemia. Neuronal cell death appeared initially in the hippocampus 3 days after ischemia and gradually increased until 7 days post-ischemia. Treatment with U0126 normalised cerebrovascular ET_B_ and 5-HT_1B_ receptor expression and contractile function, reduced hippocampal cell death and improved survival rate compared to vehicle treated animals.

**Conclusions:**

Excessive cerebrovascular expression of contractile ET_B_ and 5-HT_1B_ receptors is a delayed response to global cerebral ischemia peaking 3 days after the insult, which likely contributes to the development of delayed neuronal damage. The enhanced cerebrovascular contractility can be prevented by treatment with the MEK1/2 inhibitor U0126, diminishes neuronal damage and improves survival rate, suggesting MEK1/2 inhibition as a novel strategy for early treatment of neurological consequences following global cerebral ischemia.

## Introduction

The principal cause of global cerebral ischemia is cardiac arrest (CA), representing nearly 70% of all deaths of patients after out–of-hospital cardiac arrests. To date, therapeutic treatment of CA survivors is very poor and the subsequent global cerebral ischemia remains the major challenge to defeat [1], [2]. Imbalance between local vasodilators and vasoconstrictors, cerebral edema and blood brain barrier breakdown has been reported as contributory mechanisms of dysregulated cerebral blood flow (CBF) after global cerebral ischemia in both animals and humans [3]–[5]. It is well-established that global cerebral ischemia is associated with a post-ischemic phase of reduced CBF termed delayed postischemic hypoperfusion (PDH), which may contribute to delayed neuronal cell death where the neurons in the CA1 region of the hippocampus are particularly vulnerable, resulting in persistent cognitive deficits [6]. However, the time-course of PDH in experimental models of global cerebral ischemia is not well characterized, and the underlying molecular mechanisms are largely enigmatic. In particular, the reason for the week-long delay in the occurrence of neuronal cell death is still unclear, and a better understanding of the contributing processes taking place in the inter-rim phase between the ischemic insult and the occurrence of neurological damage is demanded.

Upregulation of vasocontractile endothelin type B (ET_B_) and 5-hydroxytryptamine type 1B (5-HT_1B_) receptors has previously been demonstrated in cerebral artery smooth muscles 48 hours after experimental global cerebral ischemia [7]. We suggest that this change in vasoconstrictor receptor expression pattern results in an increased contractile tone of the affected arteries and thereby decrease tissue perfusion contributing to delayed neuronal cell death.Therefore, we hypothesize that upregulation of vasocontractile receptors after global cerebral ischemia could be a novel target for pharmacological prevention of post-ischemic hypoperfusion and delayed neuronal death. However, the molecular mechanisms underlying this receptor upregulation have not been elucidated. We hypothesize that the receptor upregulation observed after global cerebral ischemia depends on activation of the intracellular signaling via the mitogen-activated protein kinase kinase (MEK) – extracellular signal-regulated kinase 1/2 pathway. This hypothesis is based on previous findings in experimental models of cerebral ischemia caused by different kinds of stroke [8]–[10]. In the present study the aim was to characterize the time-course of changes in ET_B_ and 5-HT_1B_ receptors in cerebral arteries in relation to the development of neuronal cell damage and neurological deficits up to seven days after transient forebrain ischemia. Moreover, the aim was to determine whether treatment with the MEK1/2 inhibitor U0126 could prevent the cerebrovascular vasoconstrictor receptor upregulation, delayed neuronal cell death and improve outcome after global cerebral ischemia.

## Material and Methods

### Animals

Animal procedures were performed strictly within national laws and guidelines and were approved by the Danish Animal Experimentation Inspectorate (licence number: 2009–1670). Wistar rats (Taconic, Denmark) weighing 250–360 g, were provided with standard rat chow and water and were housed under 12 h light and 12 h dark cycle conditions.

### *In vivo* model - Global cerebral ischemia

Rats were fasted overnight with free access to water. Reversible forebrain ischemia was induced by 15 min occlusion of both common carotid arteries combined with concomitant hypovolemia previously described by [11]. Briefly, rats were anaesthetized with 3.5% isoflurane (Abbott Laboratories) in atmospheric air/O_2_ (70:30), orally intubated and artificially ventilated with 1.5–2% isoflurane in N_2_O/O_2_ (70:30) during the surgery. Catheters were inserted in the tail artery and vein for blood pressure recording, regular blood sampling for gas analysis (Radiometer, Copenhagen, Denmark) and infusions. The body and scull temperature were continuously monitored and kept close to 37°C by a heating pad. Muscle relaxation was achieved intravenously (i.v) with Norcuron (Shering-Plough) 0.2 mg/ml, bolus dose of 0.2 ml and thereafter 1.3 ml/h to maintain paralysis. A soft polyurethan catheter filled with heparin was inserted via the external jugular vein into the right atrium. Loose ligatures were placed around each of the common carotid arteries. After completion of the surgical procedure, rats received 0.5 ml heparin (100 IU/ml) and were allowed 15–20 min of equilibration.

After equilibration, reversible ischemia was induced by lowering the mean arterial blood pressure (MABP) to 40 mm Hg by withdrawing blood through the jugular vein catheter, followed by bilateral common carotid artery clamping. After 15 min clamps were released and normal blood pressure restored by rapid reinfusion of blood. 0.4 ml 0.6 M sodium bicarbonate was injected i.v to counteract systemic acidosis. Upon removal of catheters and closure of incisions, animals were allowed to recover 15–20 min before discontinuation of isoflurane and extubation. As post operative analgesia, in the end of surgery and every 24 hours thereafter, rats were subcutaneously administered with 5 mg/kg Carprofen (Rimadyl Vet, Pfizer). In addition, fluid supplement (5 ml 0.9% NaCl isotonic solution) was administered subcutaneously when needed. Sham operated animals underwent the same surgery procedure with approximately the same surgery time, except for carotid clamping, lowering of MABP and sodium bicarbonate injection.

### Experimental design and drug administration

A total of 105 rats were used in the study. All animals were sedated with CO_2_ before they were sacrificed by decapitation. Rats were daily monitored and watched over by a well-experienced animal caretaker to avoid any visible post-surgery suffering, such as pain, low body temperature and dehydration. If any visible suffering was monitored, animals were humanely euthanized. For time-course studies 67 untreated rats were operated. Animals were euthanized at 0, 1, 3, 5 and 7 days after surgery (n = 3–14 per group and time point). In addition, 38 rats were operated for treatment studies with either 30 mg/kg 1,4-diamino-2,3-dicyano-1,4 bis [2-aminophenylthio] butadiene; LC labs, Boston, MA, USA (U0126), dissolved in 500 μl dimethyl-sulfoxide (DMSO; Sigma, St Louis, MO, USA) or 500 μl DMSO (vehicle). Rats were injected intraperitoneally (i.p) immediately after reperfusion and at 12, 24, 36, 48 and 60 h after reperfusion with either U0126 or vehicle and were then left untreated, but under daily monitoring until termination at 3 or 7 days, respectively. Treated rats in the 3 days group were randomly selected for sham-operated (n = 7), ischemia induced vehicle-treated (n = 7) or ischemia induced U0126-treated (n = 6). In the 7 day survival group rats were randomly selected for vehicle or U0126 treatment (1 sham-operated, 11 vehicle-treated, 7 U0126-treated). The used dose of U0126 was chosen based on earlier studies in a rat model of focal cerebral ischemia [9], [12].

### Neurological evaluation

#### Rotating pole test

This test evaluates the ability of the animals to traverse a horizontal pole (45 mm in diameter and 150 cm in length), which was either steady or rotating at different speeds (3 or 10 rpm). Rat performance was scored according to the score definitions described in [13].

#### Grip strength test

A grip strength meter was used to evaluate rat forelimb grip strength (in grams) as described in [14]. Two successive readings were taken for each animal.

#### Behavioural observation

An observation schedule including the following parameters was assembled: body-temperature, body-posture (curved back), eyes (closed, dry or blood), fur (piloeraction), faeces, rat noises (when handling the rat), noise sensitive, temperament (passive, aggressive), movement and balance. All 10 observations were scored as follows: normal state  =  0, medium state  =  1 and poor state  =  2. A mean score for each group was calculated for each day of observation.

### Harvest of cerebral arteries

Rats were anesthetized with CO_2_ and decapitated. Brains were removed and chilled in ice-cold bicarbonate buffer solution and the basilar artery (BA), middle cerebral artery (MCA) and the anterior cerebral artery (ACA) were isolated.

### *In vitro* pharmacology

A sensitive myograph (Danish Myograph Technology A/S) was used for recording the isometric tension in isolated cerebral arteries. Isolated vessels were cut into 1 mm long segments and mounted on two 40 μm stainless steel wires in the myograph. Measurements were recorded using a PowerLab unit and LabChart software (ADInstruments). The segments were immersed in temperature controlled buffer solution (37°C) of the following composition (mM) NaCl 119, NaHCO_3_ 15, KCl 4.6, MgCl_2_ 1.2, NaH_2_PO_4_ 1.2, CaCl_2_ 1.5 and glucose 5.5. The buffer was continuously aerated with 5% CO_2_ to maintain a pH of 7.4. Vessel segments were stretched to initial pretension of 2 mN/mm and were allowed to equilibrate for 35 min. The vessels were then exposed to a solution of 63.5 mM K^+^ obtained by partial substitution of NaCl for KCl in the previously described buffer. Only vessels responding to the K^+^ buffer by contraction of at least 2.0 mN for BA and 0.7 mN for MCA and ACA were included in the study. The presence of functional endothelium in the vessel segments from the three different treatment groups was assessed by precontraction with 5-HT followed by relaxation with carbachol. A relaxant response to carbachol was considered indicative of a functional endothelium. Concentration-response curves were obtained by concentration-contraction application of 5-carboxamidotryptamin (5-CT) (Sigma, St Louis, USA) in the concentration range 10^−12^ to 10^−5^ M and endothelin-1 (ET-1) (AnaSpec, San jose, USA) in the concentration range 10^−14^ to 10^−7^ M.

### Histological analysis

Animals were deeply anesthetized with 2.5 ml/kg of a mixture of hypnorm-midazolam (1∶1∶2) in sterile water (containing 0.079 mg/ml fentanyl, 2.50 mg/ml fluanison and 1.25 mg/ml midazolam). Rats were transcardially perfused with isotonic NaCl and fixative containing 4% paraformaldehyde in phosphate-buffered solution (PBS). Brains were removed and post-fixed in the same fixative for 4 h at room temperature and thereafter cryoprotected in sucrose solution 30% followed by 10%. Brain tissue was embedded in a gelatin medium (30% egg albumin, 3% gelatin in distilled water), frozen on dry ice and kept in –80°C. Series of coronal sections from midbrain (–3.8 mm to bregma) and forebrain (2.8 mm to bregma) were sectioned into 12 μm thick slices using a cryostat (Leica Microsystems GmBH) and thereafter processed for routinely staining with hematoxylin and eosin for microscopic examination and TUNEL staining for investigation of apoptotic cells.

### TUNEL assay

TUNEL was performed according to the manufacturer’s instructions (The DeadEnd Fluorometric TUNEL system, Promega Corporation, USA). Briefly, slides were washed in PBS 2×5 min and then permabilized in 0.2% Triton-x PBS for 5 minutes and then washed in PBS again. Proteinase-K was added for 8 min at room temperature, and after washing 100 μl equilibration buffer was added to the slides for 5–10 min. After removal of equilibration buffer, 50 μl of rTdT was added with plastic cover slips and incubated protected from light in a moisture chamber at 37 °C for 60 min. Cover slips were removed and immersed in 20xSSC (NaCl and Sodium citrate buffer), washed and mounted with DAPI/vectashield. Both negative and positive control slides were included in each individual staining procedure. PreLenS software was used for quantitative analyses.

### Immunofluorescence

MCAs were dissected out, imbedded in Tissue Tek (Gibco, Invitrogen A/S, Taastrup, Denmark) and frozen on dry ice. 10 μm thick MCA sections were cut using a cryostat (Leica Microsystems GmBH). After fixation in Stephanini’s fixative, sections were incubated with primary antibodies; sheep anti-ET_B_ (Enzo, Life Sciences) diluted 1∶100 and rabbit anti-5-HT_1B_ (Abcam) diluted 1:100, followed by incubation with the secondary antibodies Dylight 594 anti-sheep diluted 1:100 and FITC anti-rabbit diluted 1∶100 (Jackson ImmunoResearch Europe), respectively. Omission of primary antibody served as negative control, where only the autofluorescence of the lamina elastica interna was seen. Immunoreactivity was visualized at the appropriate wavelengths with a light and epifluorescence microscope (Nikon 80i; Tokyo, Japan).

### Statistical analysis

Data are presented as mean values ± standard error of the mean (SEM) and n refers to the number of rats, *p<0.05, **p<0.01, ***p<0.001. Contractility curves were statistically analyzed using 2-way ANOVA followed by Bonferroni’s posttest. Vascular contractile responses are expressed as percentage of the contractile response to 63.5 mM K^+^. E_max_ represents the maximum contraction induced by an agonist and the pEC_50_ value refers to the negative logarithm of the concentration eliciting half maximal contraction. For biphasic responses, E_max(1)_ and pEC_50(1)_ describe the high-affinity phase and the E_max(2)_ and pEC_50(2)_ describe the low-affinity phase. Significant differences in rotating pole test and grip-strength test were determined by student’s t-test comparing two groups from the same time-point. Neurological observation was analyzed by 1-way ANOVA and Student’s t-test comparing vehicle with U0126 from the same time-point. Quantification of TUNEL staining was compared by Student’s t-test.

## Results

### Animal model and physiological parameters

Physiological parameters for all rats were within acceptable physiological limits during surgery and there were no significant differences among experimental groups (Table 1). Rats that received dyscomfort from the treatment procedure were immediately terminated and excluded from the study.

**Table 1:** Physiological Parameters.

Group	MABP	MABP	MABP	pH	pH	pCO_2_	pO_2_	Body T.	CranialT.
	Preischemia	Ischemia	Postischemia	Preischemia	Postischemia	Mean	Mean	Mean	Mean
	(mmHg)	(mmHg)	(mmHg)	(kPa)	(kPa)	(kPa)	(kPa)	(°C)	(°C)
**Sham**	114±12.3	-	-	7.41±0.1	-	4.6±0.9	16.7±3.4	36.9±0.4	-
**0h**	104±10.8	39±1.4	112±10.4	7.42±0.1	7.31±0.1	5.0±0.2	15.5±1.4	36.9±0.1	37.0±0.1
**Day 1**	114±9.56	39±0.9	125±13.8	7.41±0.1	7.30±0.1	4.6±0.9	19.2±3.4	36.6±0.4	36.6±0.3
**Day 3**	105±9.94	40±1.7	116±15.4	7.39±0.1	7.30±0.1	4.9±0.7	17.3±2.4	36.6±0.2	36.7±0.3
**Day 5**	102±20.9	39±5.3	106±25.4	7.44±0.1	7.28±0.1	4.5±0.9	15.9±0.9	36.8±0.2	36.9±0.2
**Day 7**	111±17.7	40±1.9	112±14.2	7.46±0.1	7.31±0.1	4.4±0.9	15.9±2.9	36.8±0.4	37.0±0.3

The following physiological parameters were measured during surgery; mean arterial blood pressure (MABP), pH, CO_2_ and O_2_ pressure (p), body and cranial temperature (T) of control animals (sham) or animals subjected to global cerebral ischemia and reperfusion for 0, 1, 3, 5 or 7 days. Values are means ± SEM, n = 3–24 rats in each group.

### Upregulation of contractile endothelin and 5-hydroxytryptamine receptors is a delayed, transient response of cerebral arteries to global cerebral ischemia

To investigate the time-course of the earlier demonstrated enhanced contractile function of ET_B_ and 5-HT_1B_ receptors in cerebral arteries after global cerebral ischemia (Johansson *et al*., 2012), we determined the contractile responses to cumulative application of ET-1 (ET_A_ and ET_B_ receptor agonist) and 5-CT (5-HT_1B/D_ receptor-selective agonist) in the following cerebral arteries; basilar artery (BA), middle cerebral artery (MCA) and anterior cerebral artery (ACA), using a wire myograph. Potassium (K^+^, 63.5 mM) induced contraction of the smooth muscle cells was used as an internal control for normalization of agonist-induced contractile responses in each vessel segment. Mean potassium-induced responses did not differ significantly between groups.

**Figure 1:**
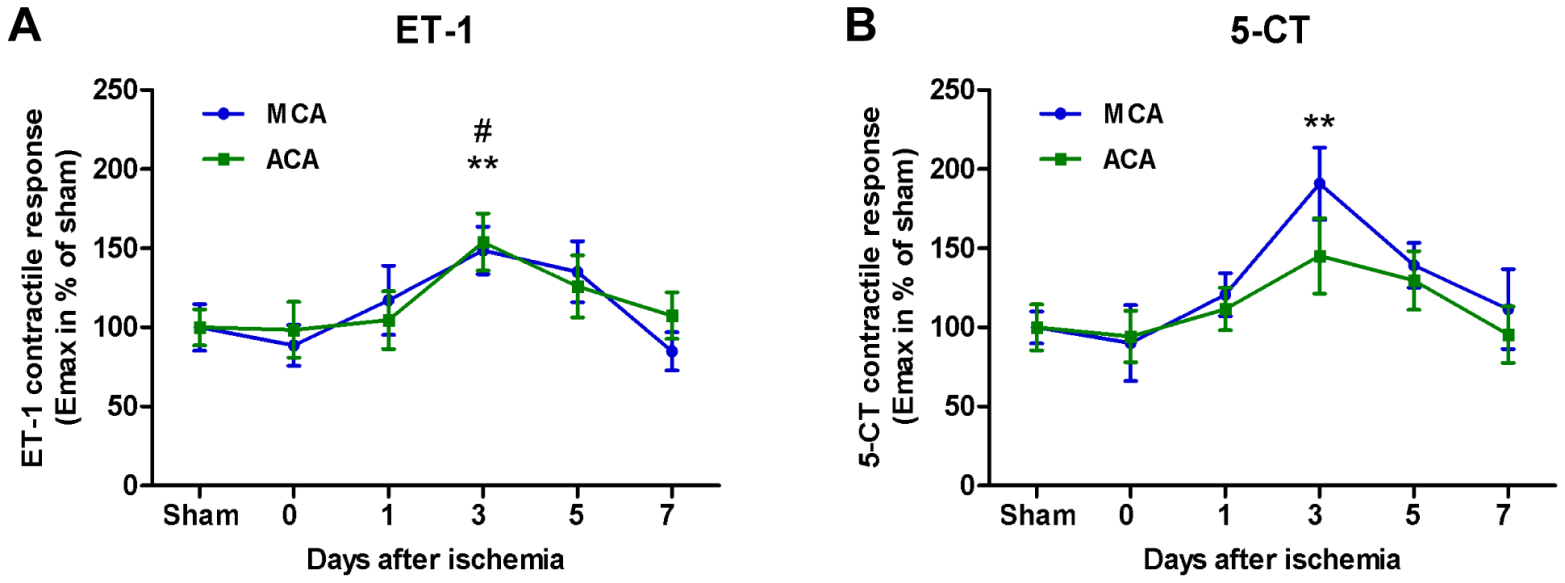
Time-course of enhanced cerebrovascular contractile responses after global cerebral ischemia. Graphs summarizing concentration-contractions curves elicited by cumulative application of ET-1 (**A**) and 5-CT (**B**) to MCAs and ACAs from control rats (sham) or rats subjected to ischemia and reperfusion for 0, 1, 3, 5 or 7 days. The maximum contraction for E_max(1)_ and E_max(2)_ were calculated for each agonist at each post-ischemic day and normalized to the corresponding sham levels and the E_max_ value on the Y axis represents a mean value received from a composition of both E_max(1)_ and E_max(2)_. n = 3–15 rats in each group. Asterisks (* for MCA and # for ACA) indicate significant differences between sham and ischemia as determined by Student's t-test.

Figure 1 summarizes data for ET-1 and 5-CT-induced contractile responses from sham-operated rats and ischemia-reperfusion rats at 0 h, 1, 3, 5 and 7 days post-ischemia.

#### Contractile responses to ET-1

In MCAs and ACAs, significantly enhanced contractile responses to ET-1 were observed at 3 days post-ischemia compared to MCAs and ACAs from sham-operated rats, whereas in 0 h, 5 days and 7 days of ischemia-reperfusion groups, the ET-1 induced contractions in MCAs and ACAs were not changed compared to sham (Fig. 1A). In BAs, contractile responses to ET-1 were unchanged at all post-ischemic time-points tested as compared to corresponding sham-operated controls (data not shown).

#### Contractile responses to 5-CT

A maximal significant contractile response to 5-CT was seen in MCAs from 3 days ischemia-reperfusion rats compared to MCAs from sham-operated rats, whereas in ACAs from 3 days ischemia-induced rats the contractile response to 5-CT was not significantly enhanced compared to ACAs from sham-operated animals (Fig. 1B). In MCAs and ACAs from 0 h, 5 days and 7 days of ischemia-reperfusion rats, contractile responses induced by 5-CT were unchanged compared to sham-operated rats (Fig. 1B). In BAs, the contractile responses evoked by 5-CT were unchanged at all time-points by the ischemia (data not shown).

In summary, the enhanced contractile responses in MCAs and ACAs evoked by ET-1 and 5-CT showed a delayed time-course peaking by day 3 post-ischemia and had a transient nature being fully reversed at day 7 post-ischemia.

### Sensorimotor deficits develop early after global cerebral ischemia

**Figure 2:**
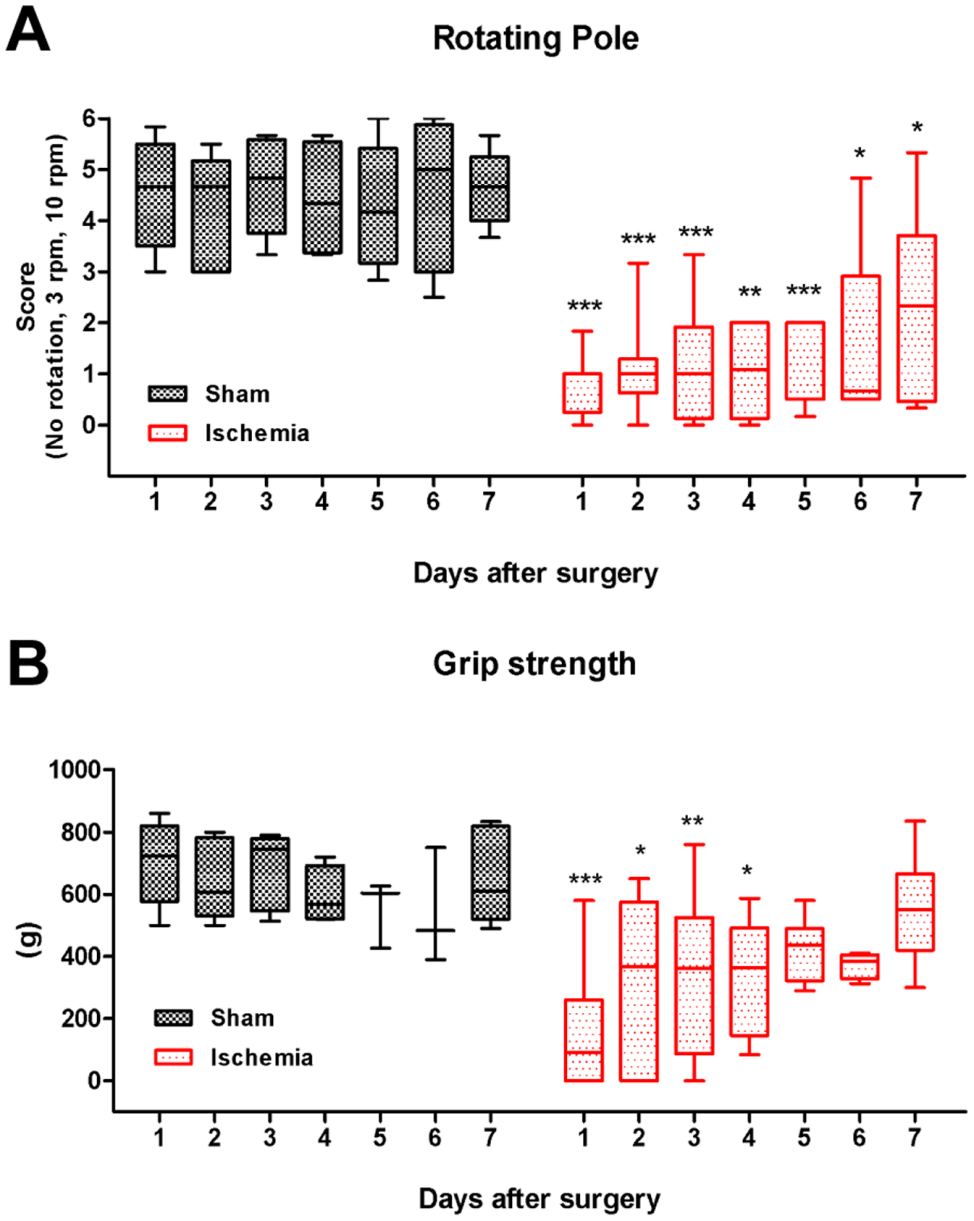
Time-course of sensorimotor deficits. Rotating pole test (**A**) and grip strength test (**B**) was performed each day up to 7 days after sham-operation or global cerebral ischemia. All data are means ± SEM, n = 7–16 number of rats. Stars indicate significant differences between sham-operated rats compared to corresponding (same day after surgery) ischemia-operated rats as determined by Student's t-test. *p<0.05, **p<0.01, ***p<0.001

To determine the time-course of development of sensorimotor deficits after the ischemic insult, rats were assessed using two different neurological tests; a rotating pole- and grip strength test at day 1, 2, 3, 4, 5, 6 and 7 after the ischemic insult (Fig. 2). In the rotating pole test, rats scored significantly worse than sham-operated rats at all time points. At day 6 and 7 a tendency to recovery was observed and the rats were able to balance on the pole. However, they still scored significantly lower than the corresponding sham rats (Fig. 2A). In the grip strength test, ischemia rats performed significantly worse the first days compared to sham but then gradually recovered and at day 7 the difference was not significant (Fig. 2B).

### Delayed neuronal cell death and mortality after global cerebral ischemia occurs at post-ischemic time-points later than upregulation of cerebrovascular constrictor receptors

**Figure 3:**
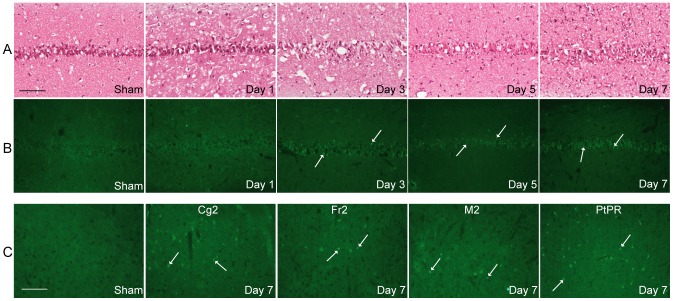
Time-course of neuronal cell death after global cerebral ischemia. Representative microphotographs of HE staining (**3A**) and apoptotic DNA fragmentation detected by TUNEL staining (**3B**) in hippocampus CA1 area from sham operated rats or ischemia induced rats at 1, 3, 5 or 7 days of reperfusion. (**3C**) shows microphotographs of apoptotic DNA fragmentation detected by TUNEL staining in cortex from sham-operated and ischemia-induced rats 7 days after surgery. (**3A and B**) shows hippocampal CA1 neurons from sham-operated rat, ischemia-reperfusion after 1 day, ischemic hippocampal CA1 neurons from day 3 after surgery, ischemia rat at day 5 and hippocampal pyramidal cells from ischemia-induced rat a week after surgery. Scale bar (100 μm) in 3A (sham) applies to all figures (3A and B), magnification of 20x. (**3C**) shows frontal cortex area 3 (Fr3) from sham-operated rats, cingulate cortex area 2 (Cg2) from ischemia- induced rat, Fr3 from ischemia-induced rat, secondary motor cortex (M2) from ischemic rats and parietal cortex, posterior area, rostral part (PtPR) from ischemia-induced rats. Scale bar (100 μm) in 3C (sham) applies to all microphotographs from cortex, magnification of 20x. White arrows are pointing at TUNEL positive cells. n = 3 rats in each group.

To characterize the time-course of neuronal cell damage induced by ischemia, routine hematoxylin eosin and TUNEL staining of brain sections were assessed (Fig. 3). Delayed neuronal damage in the hippocampus CA1 area was apparent from three days post-ischemia and was widespread after seven days (Fig. 3A and B). The morphology of the CA1 pyramidal neurons three days after ischemia showed vacuolation and cells appeared slightly shrunken (Fig. 3A). At days five to seven neurons became gradually more pyknotic with a densely stained shrunken appearance with minimal cytoplasm (Fig. 3A). TUNEL positive cells were observed in the hippocampus CA1 area three days after ischemia (Fig. 3B). Furthermore, even more widespread TUNEL positive cells were found in the same area at day 5 and 7 (Fig. 3B). In contrast, no morphological changes or TUNEL positive neurons were seen in sham-operated animals or at one day after ischemia (Fig. 3A and B).

TUNEL positive cells were observed in patches in for- and midbrain cortex (bregma 2.8 and –3.8 respectively), but were mostly pronounced in areas such as cingulate cortex area 2 (Cg2), frontal cortex area 3 (Fr3), secondary motor cortex (M2) and in parietal cortex, posterior area, rostral part (PtPR) after day 7 (Fig. 3C).

Interestingly, a delayed post-ischemic mortality was observed showing a similar time-course as the delayed neuronal damage. Thus, 4 out of 12 rats died suddenly in the group intended to survive until 7 days post-ischemia (at 5 and 6 days post-ischemia), whereas no rats died in the other groups.

### Treatment with MEK1/2 inhibitor U0126 prevents upregulation of cerebrovascular contractile receptor function and expression

To determine the effect of U0126 treatment on ischemia-induced upregulation of vasoconstrictor receptors at a functional level in cerebral artery smooth muscle cells, contractile responses to ET-1 and 5-CT were investigated in MCAs and ACAs at 3 days post-ischemia compared to sham-operated rats using a wire myograph. For normalization of the contractile responses evoked by ET-1 and 5-CT, 63.5 mM potassium-induced contraction for each vessel segment were used as an internal control (set to 100%). Mean potassium-evoked responses did not differ significantly between the groups (*Table 2*). All segments displayed a functional endothelium and there were no significant differences between the three groups (data not shown).

**Table 2:** Inhibition of cerebrovascular contractile responses by U0126.

Group	n	K^+^ response	E_max(1)_ (%)	E_max(2)_ (%)	pEC_50(1)_ (-log M)	pEC_50(1)_ (-log M)
**ET-1 MCA**						
Sham	6	3.42±0.50	20.7±4.93*	135.0±11.3*	–11.3±0.43	–8.69±0.12
Vehicle	6	2.41±0.36	44.7±11.7#	191.0±19.3#	–11.5±0.52	–9.16±0.23
U0126	6	3.18±0.36	12.6±6.03*	146.8±9.30	–11.8±1.16	–8.87±0.11
**ET-1 ACA**						
Sham	5	3.21±0.51	36.6±9.94	134.2±20.1	–11.7±0.53	–9.09±0.25
Vehicle	6	3.46±0.54	65.0±13.1	140.2±14.9	–12.3±0.40	–9.63±0.28
U0126	6	4.10±0.36	31.8±17.4	124.5±7.21	–12.6±0.78	–9.33±0.21
**5-CT MCA**						
Sham	6	3.42±0.50	31.5±7.60*	89.17±10.6*	–7.77±0.26	–5.17±0.28
Vehicle	6	2.41±0.36	61.7±12.3#	140.8±15.0#	–7.78±0.22	–5.13±0.28
U0126	6	3.18±0.36	26.3 ±7.46*	89.83±9.35*	–8.32 ±0.42	–5.37±0.20
**5-CT ACA**						
Sham	5	3.21±0.51	39.2±3.45	113.0±10.6	–7.91±0.24	–5.38±0.18
Vehicle	6	3.46±0.54	47.8±11.7	114.3±14.5	–8.02±0.37	–5.77±0.30
U0126	6	4.10±0.36	22.3±6.63	94.50±16.4	–8.49±0.79	–5.65±0.18

Pharmacological parameters for contractile responses elicited by ET-1 and 5-CT in middle cerebral artery (MCA) and anterior cerebral artery (ACA) 3 days after surgery in sham-operated rats and ischemia-induced rats treated with either U0126 or vehicle. The contractile response curves were characterized by maximum contractile response (E_max1_ and E_max2_) values, expressed as percentage of 63.5 mM K^+^ induced contraction (K^+^ response), and values for the negative logarithm of the molar concentration that produces half maximum contraction (pEC_50_), for each of the two phases respectively. Values are means ± SEM, n  =  numbers of rats. * indicates significant differences from vehicle (ischemia + vehicle treatment) and # indicates significant differences from sham (sham-operated), determined by Student’s t test comparing (E_max_) values between two groups.

#### Contractile responses to ET-1

**Figure 4:**
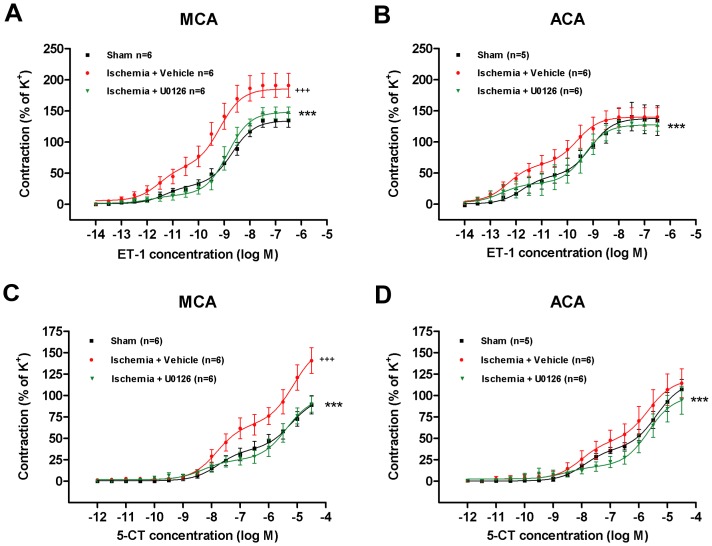
Effects of U0126 on enhanced vasoconstriction. Graphs showing concentration-contraction curves for MCAs (**A and C**) and ACAs (**B and D**) stimulated with cumulative doses of ET-1 (**A and B**) or 5-CT (**C and D**) obtained 3 days post-surgery. Data show sham-operated rats (sham), ischemia-induced rats treated with vehicle (ischemia + vehicle) and ischemia-induced rats treated with U0126 (ischemia + U0126) (30 mg/kg). Values are expressed as means ± SEM in percentage of contractions evoked by 63.5 mM of K^+^. Crosses indicate significant differences between sham and vehicle-treated ischemia-induced animals as determined by 2-way ANOVA. Stars indicate significant differences between U0126- and vehicle-treated ischemia induced rats as determined by 2-way ANOVA. n = 5–6 rats in each group. ***p<0.001

In MCAs and ACAs from ischemia-reperfusion rats treated with vehicle, the ET-1 induced concentration-contraction curves were leftwards shifted with significantly enhanced E_max1_ and E_max2_ as compared to sham. These contractile responses have previously been characterized in detail using specific antagonists to reflect two different ET-1 receptor subtypes, ET_A_ and ET_B_, in the smooth muscles of MCAs and ACAs after global cerebral ischemia [7]. Treatment with U0126 (30 mg/kg) of ischemia-induced rats resulted in a rightward shift of the ET-1 concentration-contraction curves for MCAs and ACAs back to the level of the curves for sham-operated rats. This indicates that U0126 prevents the ischemia-induced increased functional vasoconstriction mediated by ET-1 (Fig. 4A and B).

#### Contractile responses to 5-CT

5-CT evoked biphasic concentration-contraction curves in MCAs and ACAs from sham-operated and ischemia-induced rats respectively, indicating the presence of two 5-HT receptor subtypes, as demonstrated previously [7]. The contractile responses evoked by 5-CT in MCAs from ischemia-induced rats (vehicle treated) were observed as leftwards shift of concentration-contraction curves with significantly enhanced E_max1_ and E_max2_ values as compared to sham (Fig. 4C). In ACAs from vehicle treated ischemia-induced animals, no elevated contraction was observed compared to sham (p>0.05) (Fig. 4D). However, treatment with U0126 (30 mg/kg) prevented this functional 5-HT receptor upregulation in MCAs and ACAs respectively, resulting in a rightwards shift of the curves to sham levels (Fig. 4C and D).

### ET_B_ and 5-HT_1B_ receptor protein expression

**Figure 5:**
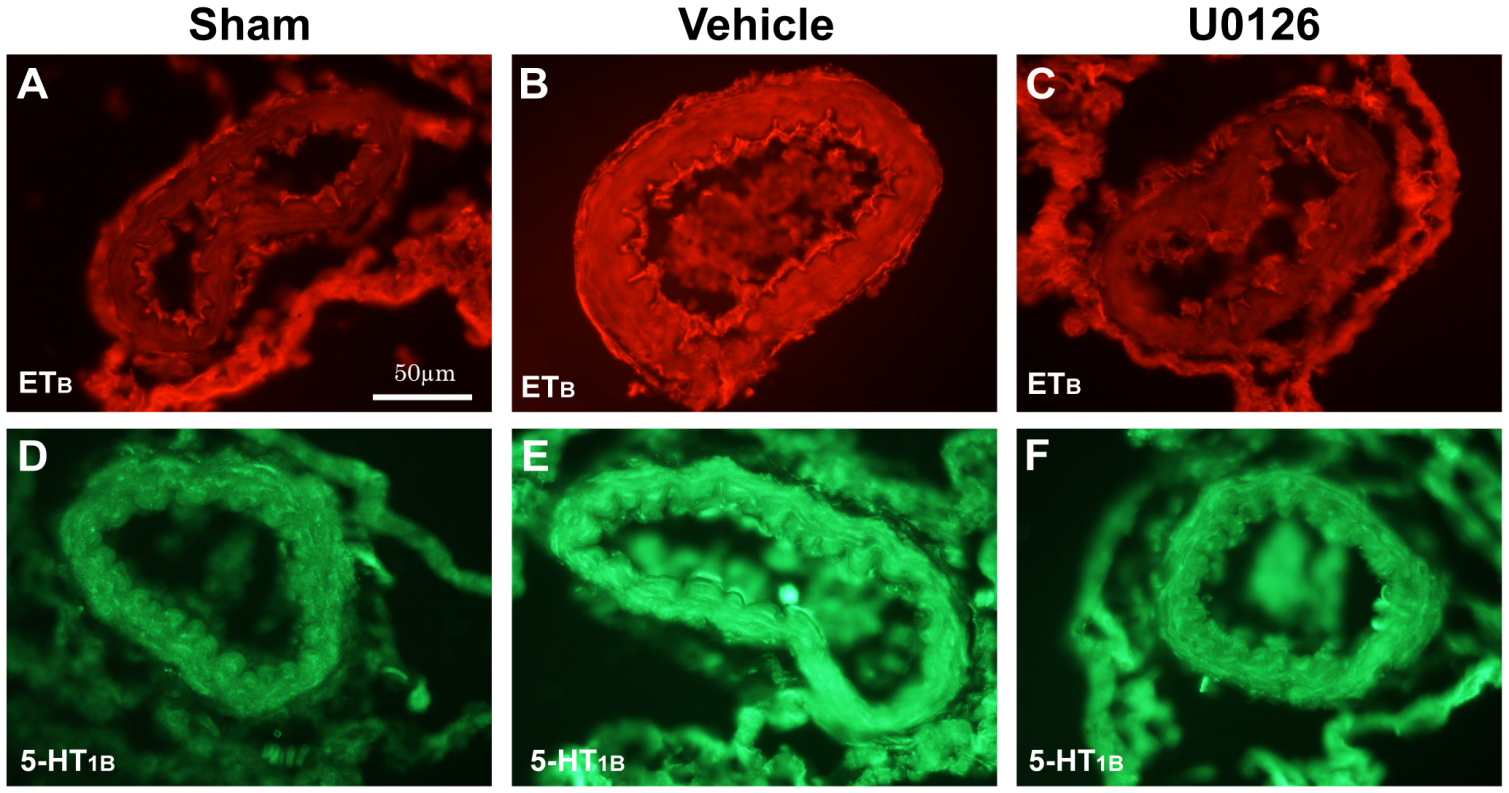
Effects of U0126 on enhanced vasoconstrictor receptor expression. ET_B_ and 5-HT_1B_ receptor expression were determined by immunofluorescence staining in the smooth muscle cells of MCAs 3 days after surgery. Representative photomicrographs presented for sham-operated rats were shown in (**A and D**), ischemia-induced rats treated with vehicle in (**B and E**) and for ischemia-induced rats treated with U0126 in (**C and F**). The upper row are showing red fluorescent staining with antibodies against ET_B_ receptor (**A-C**) and in the lower row photomicrographs showing green fluorescent staining with antibodies against 5-HT_1B_ receptor (**D-F**). Three MCA sections from each group were analyzed and one representative image for each group is shown. n = 6-7 rats in each group. Scale bar in A applies to all figures, magnification of 40x.

To determine the receptor localization and investigate the effect of U0126 treatment on ischemia-induced upregulation of smooth muscle vasoconstrictor receptor expression we performed immunofluorescence with antibodies against ET_B_ (Fig. 5A-C) and 5-HT_1B_ (Fig. 5D-F) receptors in MCAs from sham-operated rats and ischemia-induced rats treated with vehicle or U0126. In consistency with the earlier study in global cerebral ischemia, which has been confirmed and quantified using Western blotting [7], we observed an increased expression of these receptors in the smooth muscle cells of arteries from ischemia-induced rats (vehicle treated) compared to sham (Fig. 5A, B, D and E). Moreover, we observed that treatment with U0126 suppressed the increased ET_B_ and 5-HT_1B_ receptor expression in the smooth muscle cells to sham-levels (Fig. 5C and F).

### U0126 prevents delayed neuronal cell death after global cerebral ischemia

**Figure 6:**
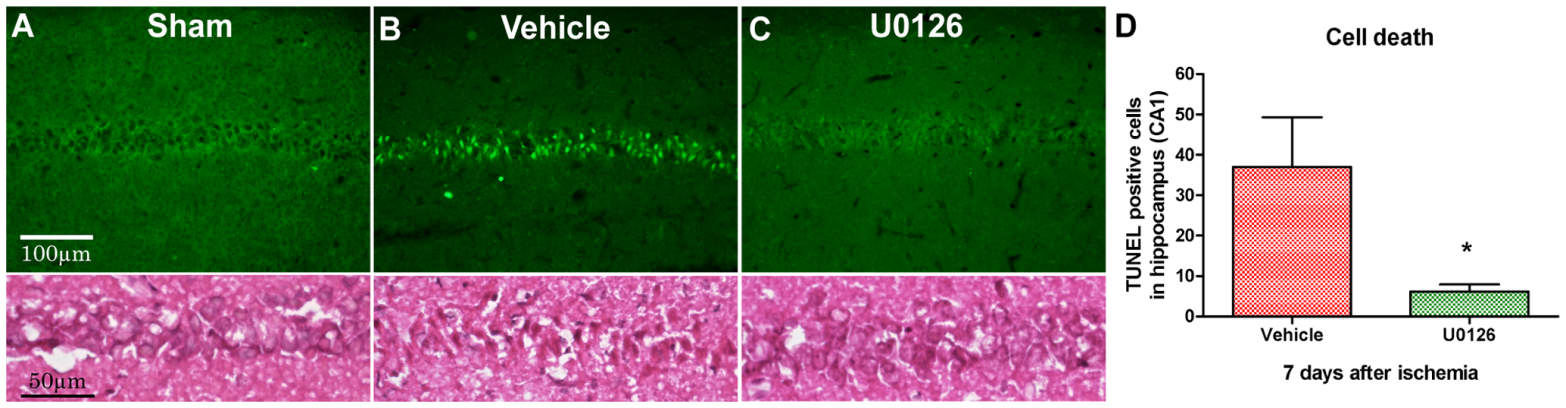
Effects of U0126 on delayed neuronal cell death. Representative microphotographs of TUNEL staining (apoptotic DNA fragmentation detection) show the effect of U0126 on the neuronal damage of hippocampal CA1 neurons, in rats 7 days after surgery (**A-C**). (**A**) TUNEL staining of coronal sections from sham-operated rats. (**B**) TUNEL positive cells in the vehicle treated group. (**C**) U0126 group. (**D**) Bars are illustrating quantitative measurements of U0126 effects on CA1 hippocampus neuronal apoptosis 7 days after ischemia, showing significant decreased number of TUNEL positive cells in U0126 compared to vehicle treated animals. Values are means ± SEM, n = 5–6 numbers of rats in each group. Significant differences were determined using Student's t-test. *p<0.05.

To investigate the effect of U0126 treatment on delayed neuronal cell death following global cerebral ischemia, TUNEL staining was performed on midbrain coronal sections (-3.8 to bregma) to detect DNA damage of hippocampal CA1 pyramidal neurons 7 days after reperfusion (Fig. 6). In the CA1 region from control rats (sham-operated) normal intact neurons were revealed (Fig. 6A). However, in the hippocampal CA1 area from ischemia vehicle treated animals (n = 5) obvious neuronal damage was observed, as indicated by increased numbers of TUNEL positive cells compared to sham-operated (Fig. 6B). Furthermore, delayed neuronal death was significantly reduced in U0126 treated animals (n = 6) as compared to vehicle treated animals (Fig. 6C and D).

### U0126 improves outcome and survival rate after global cerebral ischemia

**Figure 7:**
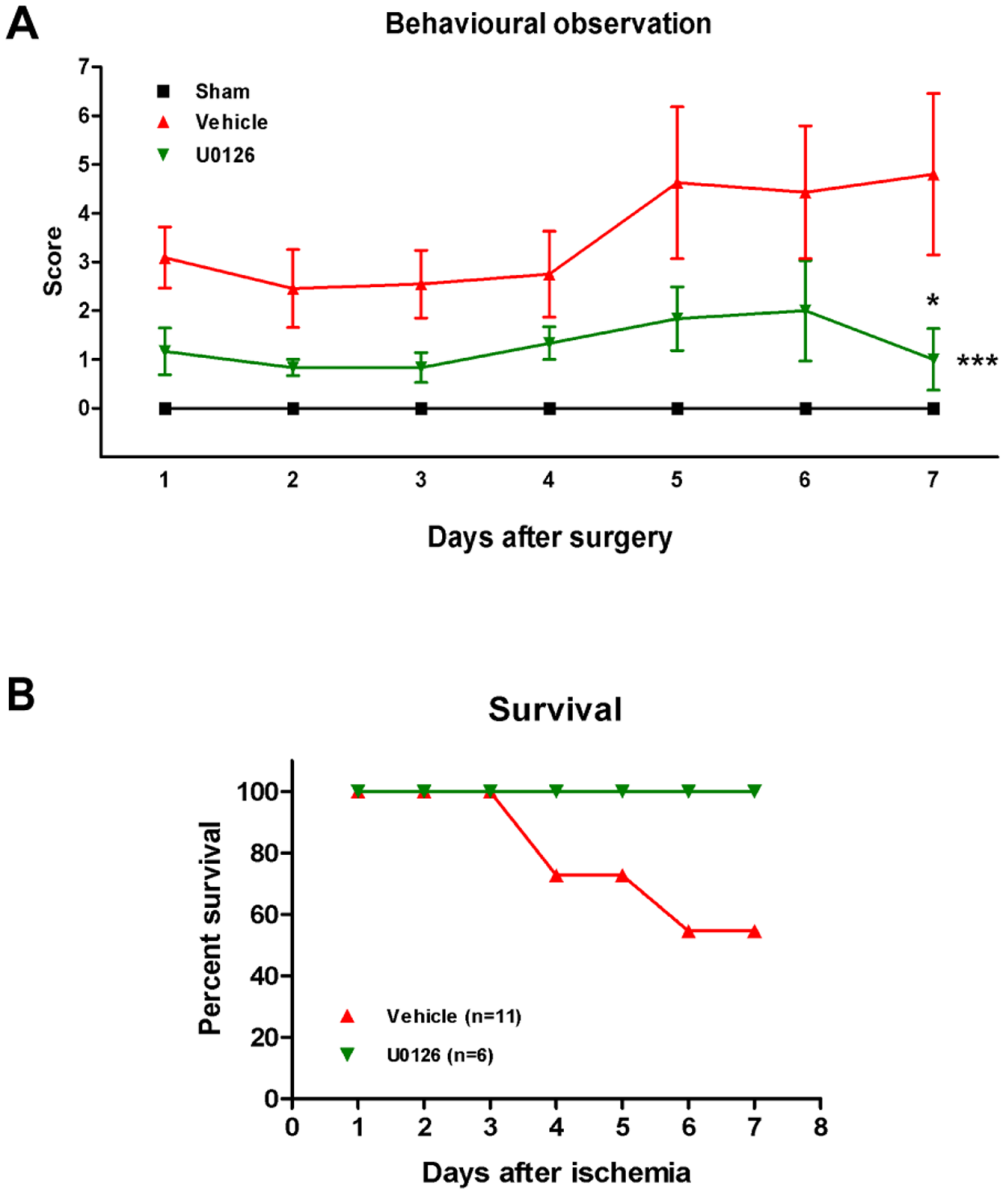
Effect of U0126 on outcome. Graphs demonstrating neuronal observation of rat behavioral pattern (**A**) and survival rate (**B**) 1–7 days after rats were subjected to sham-operation or ischemia with either treatment of vehicle or U0126. (**A**) Ten different parameters of neurological observations were translated into scores, where 0 points indicated a normal behavioral pattern. *** on the right side indicate significant differences between vehicle and U0126 as determined by 1-way ANOVA. * above the error bar indicate significant differences between vehicle and U0126 determined by Student's t-test comparing rats from the same day. Values are means ± SEM, n = 5–11 numbers of rats in each group. *p<0.05, ***p<0.001. (**B**) Graph showing rat survival rate for ischemia-induced rats treated with either vehicle or U0126 up to a weak after induced ischemia. Rats in the U0126 group have a 100% survival rate whereas vehicle treated rats died in the subacute phase of ischemia, showing only 55% survival rate. n = 6–11 numbers of rats in each group.

The two neurological tests used to characterize the time-course of sensorimotor deficits after global cerebral ischemia (rotating pole and grip strength) appeared not to reflect the delayed processes relevant to U0126 action (data not shown). Hence, a neurological observation schedule was assembled to capture any possible effect of U0126 treatment on neurological deficits, abnormal behavioral pattern and general well-being of the animals after global cerebral ischemia (Fig. 7).

Observations of rat body-temperature, body-posture, eyes, fur, feces, rat noises, noise sensitivity, temperament, movement and balance were performed daily by an observer that was blinded to the identity of study groups. The sham-operated rat (used as a control) accomplished score 0 all days after surgery, indicating a normal rat neurological and behavioral profile and a general well-being. Vehicle treated animals acquired noticeable higher scores compared to U0126 treated rats already in the acute post-ischemic phase but the difference did not become significant until day 7 post-ischemia, reflecting delayed full effect of the U0126 treatment on outcome (Fig. 7A).

Interestingly, treatment with U0126 also improved 7 days survival rate of the animals. Thus, all rats treated with U0126 survived 7 days post-surgery, corresponding to a 100% survival rate. However, in a group of 11 animals, 5 vehicle-treated rats died suddenly, before reaching day 7, which is equivalent to a survival rate of merely 55% (Fig. 7B).

## Discussion

The present study reveals a central mechanism behind cerebrovascular upregulation of vasoconstrictor receptors, a recently discovered consequence of global cerebral ischemia. We demonstrated for the first time that the excessive vasoconstrictor receptor expression is a transient subacute reaction of the cerebral vasculature to the ischemic insult, preceding the development of delayed neuronal damage. We also showed for the first time that the enhanced ET-1 and 5-HT evoked receptor mediated cerebrovascular contractility, presumably involved in delayed cerebral hypoperfusion after global cerebral ischemia, can be prevented by treatment with an inhibitor of MEK/ERK1/2 signaling. Interestingly, this treatment also diminished neuronal hippocampal CA1 damage and improved rat neurological outcome and survival rate, suggesting that prevention of cerebrovascular receptor changes via inhibition of this central signaling pathway is a novel therapeutic approach to prevention of cerebral hypoperfusion and resulting brain damage after global cerebral ischemia.

It is well documented [11], [15]–[17], and confirmed in the present study, that global cerebral ischemia is associated with a delayed type of neuronal cell death, principally affecting the hippocampal CA1 pyramidal cells in the subacute phase (from 3 days after the insult) and subsequently reaching a maximum after a week. Moreover, we demonstrate less severe cell death in the prefrontal neocortex, a finding that is also in accordance with earlier studies [18]. The source leading to development of delayed neuronal damage after global cerebral ischemia is not clearly particularized, but several studies have emphasized the importance of delayed cerebral perfusion disturbances, indicating a correlation between reduced local cerebral blood flow and selective vulnerable neuronal cell death [19]–[21]. The most well-described cerebral perfusion deficit associated with global cerebral ischemia is delayed post-ischemic hypoperfusion, which may last as long as several days after the ischemic insult [22]–[25]. Longer durations of global cerebral ischemia have been demonstrated to increase the severity of post-ischemic hypoperfusion [26], in contrast to the severity of post-ischemic hypoperfusion, it has been argued that the longer the ischemia lasts the later the onset of the hypoperfusion [27]. Thus, delayed post-ischemic hypoperfusion appears to be a central part of the pathophysiology underlying neuronal cell death after global cerebral ischemia.

However, the pathophysiological mechanisms behind delayed hypoperfusion have not been disambiguated. Factors proposed to be involved are vascular vasospasms and increased plasma and brain levels of vasoactive substances such as endothelin-1 and 5-hydroxotryptamine. Studies of global cerebral ischemia after cardiac arrest in cats demonstrated alterations in vasoconstrictive responses 24 hours following global cerebral ischemia, suggesting that the delayed hypoperfusion is associated with vasoconstriction and/or vasospasm of the cerebral vasculature [28]. Besides, endothelin-1 has been shown to be elevated in the post-arrest period in cardiac arrest patients and in the delayed hypoperfusion period after global cerebral ischemia in dogs [3], [5]. Twenty hours after induced unilateral ischemia in Mongolian gerbils, 5-hydroxytryptamine was significantly increased in the brain tissue [29].

Here we demonstrated a transient increased vasocontractile response mediated by ET-1 and 5-HT receptors in cerebral arteries in the subacute phase after global cerebral ischemia, leading to increased contractile tonus in the cerebral vasculature and likely contributing to delayed hypoperfusion. We have earlier demonstrated that the increased delayed vasoconstriction after global cerebral ischemia is associated with upregulated ET_B_ and 5-HT_1B_ receptors in the smooth muscle cells of cerebral arteries [7]. In freshly isolated cerebral arteries from healthy animals, ET_B_ and 5-HT_1B_ receptors have been shown to be expressed in the endothelial cells [30]. However, we did not find a change in their endothelial expression following global cerebral ischemia.

We investigated the time-course of the enhanced endothelin-1 and 5-hydroxytryptamin mediated contraction in cerebral arteries, to find out whether the enhanced cerebral vasoconstriction precedes the appearance of delayed neuronal damage in time. Our results showed that the receptor upregulation peaks in the inter-rim period between the ischemic insult and the occurrence of neuronal damage. Although we are aware that this correlation does not necessarily imply a causal relationship between the upregulated vasoconstrictor receptors and delayed hypoperfusion and neuronal damage, the time-courses of these events suggest that the enhanced vasoconstriction could be a contributing factor to the development of delayed neuronal damage by decreasing perfusion of the brain tissue in the subacute phase after the insult.

Current possibilities for pharmacological interventions of global cerebral ischemia are very poor and today the only available treatment is mild therapeutic hypothermia, which has recently become a standardized intervention after cardiac arrest [31], [32]. Despite the improvement made by therapeutic hypothermia, the poor survival rate of out-of-hospital cardiac arrest patients remains around 8%, which makes the unmet need for effective pharmacological therapeutic approaches still urgent [33], [34]. A number of specific neuroprotective agents have proven to be effective in experimental models of global cerebral ischemia, but when advanced into clinical trials, they failed to bring about significant improvements [35]. Only very few pharmacological agents targeting the cerebrovascular pathophysiology in the post-ischemic period after global cerebral ischemia have hitherto been explored [36].

This is the first study to demonstrate that the delayed increased cerebrovascular vasoconstrictor ET_B_ and 5-HT_1B_ receptor upregulation in cerebral arteries after global cerebral ischemia can be abolished by early post-ischemic treatment with the specific MEK1/2 inhibitor U0126. This suggests that inhibition of this signaling pathway could be a novel pharmacological strategy against delayed post-ischemic hypoperfusion. Interestingly, treatment with this compound was not only able to prevent vasoconstriction, but also diminish delayed hippocampal CA1 neuronal damage, improve neurological outcome and prodigiously, also improve rat survival rate. Although the neuroprotective effect of U0126 have recently been suggested to be due to direct effects of U0126 on the neurons following global cerebral ischemia in rats [37], [38], we suggest based on the findings in the present study, that U0126 exerts its beneficial effects, at least in part, via its effects on the vasculature: prevention of vasoconstrictor receptor upregulation leading to increased cerebral perfusion and thereby less delayed neuronal damage.

In conclusion, the findings of the current study demonstrate a delayed enhanced expressional response of vasocontractile receptors to global cerebral ischemia peaking three days after the insult, resulting in increased cerebrovascular contractility, which may contribute to delayed post-ischemic hypoperfusion and development of delayed neuronal damage. Furthermore, the altered cerebrovascular contractility can be prevented by treatment with the MEK1/2 inhibitor U0126, diminish neuronal damage and improves survival rate. These results suggest that MEK1/2 inhibition could be a novel pharmacological approach for early treatment of the detrimental secondary consequences following global cerebral ischemia.
